# Practice of Medicine

**Published:** 1859-11

**Authors:** 


### PRACTICE OF MEDICINE.

1. CASES OF CHOREA TREATED BY SULPHATE OF ZINC. By *William H. Stone, M.B.*, Oxon., *L.R.C.P.*—Fifty-four cases of chorea were admitted into St. Thomas's Hospital during the year 1858. From the similarity of conditions under which they occurred, they offer a fair opportunity for comparing different methods of treatment. They have accordingly been collected with some more detail than could be attempted in the registrar's statistical report; while, on the other hand, no attempt has been made to give the whole clinical history. Only so much has been preserved as may show the general type and causation of the disorder in the several cases, and tend to establish the efficacy of various medication.

Out of the whole number, fifty were put under the influence of three principal remedies, either singly or consecutively. In sixteen of these, the treatment mainly comprised the exhibition of sulphate of zinc; in twenty, of arsenite of potash; in fourteen, of ferruginous preparations. The remaining four cases have some exceptional modification. It is proposed in this communication to analyze briefly the group of cases treated by sulphate of zinc, and to compare them with the results obtained by other methods:—

*Case* 1.—M. D., aged sixteen, female servant. Catamenia regular and somewhat profuse. In other respects health good. Nine weeks before, after a fall, began to suffer from chorea, for which she was out-patient at Guy's Hospital. On admission, not a very severe case; speech and swallowing unaffected; some constipation and headache; aspect anæmic. Never had rheumatism. Took zinci sulph. gr. ij. t. d. for seven days; gr. iv. for seven days, but without much improvement. There was then some spinal tenderness, and a blister was made with liq. vesicatorius over the tender spot, with advantage. She then began to take ferri ammon. citr. gr. x. t. d., and a few days later a daily shower-bath. Under this treatment she rapidly improved, and on the twenty-sixth day of its administration was presented cured, having been in the hospital altogether forty-two days.

*Case* 2.—T. F , aged fourteen, a female, at home. Chorea one month, entirely limited to right side of body; no cause known; suffers much from headache. Some evidence of commencing menstruation, but no complete establishment of the function. Appetite and digestion good; no difficulty of swallowing; speech had been affected at first, but was not so on admission. No history of rheumatism; no cardiac symptoms. Took zinci sulph. gr. ij. t. d. for three days; then gr. iv. for fifty-three days. Occasional purgatives, and a shower-bath daily. Made a slow, but steady recovery in fifty-six days.

*Case* 3.—M. L. D., aged eleven, female, at home. Always a feeble child. Two months previously had some sort of febrile attack, with engorged glands in neck, and herpetic eruptions round the mouth. On recovering from this, was remarked to have choreic symptoms. These had been but slight until five days before admission; they then increased so much as to cause her to fall down, and to interfere much with articulation. Never had rheumatism. On admission she complained principally of weakness and vertigo; no headache; tongue spasmodically thrust out, and speech difficult, but no dysphagia. No cardiac symptoms; no digestive disturbance.

Took zinci sulph. gr. ij. t. d., in infusion of valerian for seven days. The dose was then increased to gr. iij. for three days, then to gr. v. for seven days, then to gr. vj. for eleven days. With this were ordered shower-baths on alternate days, and occasional purges. She made some improvement, but at the end of twenty-eight days was far from cured. She was then ordered liq. arsenicalis ♏ij. t. d., c. decoct. cinchonæ, under which treatment the improvement increased rapidly, and after taking this for thirty days, she was presented cured, having been in the hospital altogether sixty days.

*Case* 4.—H. C., aged eleven, male, at home. A very slight case; no assignable cause; general aspect and functions tolerably healthy; had been ill three weeks. Took zinci sulph. gr. ij. t. d. ex infus. valerianæ for seven days, increased to gr. iv. for two days; but as this quantity caused some sickness, it was again reduced to gr. iij., which was taken for twenty-two days, with steady improvement. He went out cured at the end of thirty-one days. Besides the zinc, a daily shower-bath was given, and occasional purgatives.

*Case* 5.—G. C., aged twelve, male, at home. Five weeks previously had been in Guy's Hospital, under Dr. Owen Rees, with rheumatic fever. There had been no cardiac symptoms or treatment. He left the hospital pretty well fourteen days previously, and choreic symptoms came on about a week before admission into St. Thomas's. There was no fright or other cause. On coming in there was some dysphagia, much difficulty in speech, rapid and spasmodic protrusion of tongue. He complained of no pain, but was kept awake at night by the movements. These increased somewhat at first, and were accompanied by stupor and drowsiness. A fortnight later the spinal muscles became firmly contracted, so as to lift him from the bed, and there was then, for the first time, spinal tenderness in the interscapular region. This subsequently became very marked at the lower part of the lumbar region. A week later the choreic symptoms abated slightly; but tenderness became marked in the cardiac region, with much anxiety of aspect, and a to-and-fro sound was audible over the same spot. Pulse 136, large, but compressible; cough becoming troublesome. Soon afterwards inability to lie on the left side, with short breath, set in, the heart's action having become irregular.

Six weeks after admission the chorea was almost gone. Pulse 132, regular. From this time he improved rapidly, though exceedingly anæmic. There remained, however, some thickening and tenderness about many of the larger joints.

At the end of three months he was able to go out, in tolerable health, but with a persistent systolic mitral murmur, and increased impulse over the cardiac region.

On first admission, he took zinci sulph. gr. ij. t. d. for ten days; then gr. iij. for fourteen days; then gr. iv. for fourteen days. At this time spinal tenderness was treated by a blister over the spot, and the syrup ferri iod. was given in ʒss. doses thrice a day. With this he improved in nervous symptoms, but the heart became affected. After taking the syrup for twenty-five days, the compound iron pill was substituted for eight days, and a mixture of quinine and iron for the three weeks before his going out. He was altogether fifty-six days under treatment. This patient returned to the hospital a few weeks later with symptoms of cardiac disease, of which he died. On post-mortem, extensive cardiac mischief, principally involving the mitral valve, was found. The spine exhibited no morbid appearances.

*Case* 6.—E. H., aged sixteen, female, at home. Catamenia had appeared more than two years before, and had been fully established above a year. Had suffered from slight choreic symptoms rather over twelve months. They began in the head and neck, but had never been very severe. No cause could be assigned for the attack; speech, swallowing, and protrusion of tongue were normal; general aspect anæmic; no rheumatism or cardiac disease. The sulphate of zinc gr. ij. t. d. ex infus. valerianæ was given for seven days, then gr. iij. for seven days, then gr. iv. for thirty-eight days. Tepid shower-bath on alternate days; and latterly aloetic purges were added from menstrual insufficiency with constipation. Under this treatment she steadily improved, and was presented, after fifty-two days, in a fair way of recovery, though not quite cured.

*Case* 7.—A. W., aged twelve, female. A feeble child; no appearance of catamenia; always nervous, and easily frightened; never had rheumatism; four days before admission frightened by "a ghost;" fell down and had spasmodic movements of the right side of the body. They had somewhat increased, but were not very severe on admission. The bowels were confined; tongue foul; much nausea and retching, but no worms known of; latterly had been subject to short breath, and palpitation on slight exertion. At these times the cardiac action was excessive, but without definite murmur. She took zinci sulph. gr. ij. ter die, ex infus. valerianæ for seven days, with shower-bath daily, and occasional purges. But it caused so much disturbance of the stomach, already irritable, that it was exchanged for the liq. arsenicalis ♏ iij. ter die c. decoc. cinchona, with free purgation. This was taken for fifteen days with ease, and rapid amendment. She went out cured after twenty-five days.

*Case* 8.—S. A., aged twelve, female. A second attack. The first had been slight; this was rather severe. It dated from a fright three weeks previously. No great affection of speech, more of swallowing. Tongue steadily protruded. General health good. Never had rheumatism. Took zinci sulph. gr. ij. t. d., ex inf. valerianæ for three days; then gr. iv. for twelve days, with daily shower-bath. Recovered rapidly. Presented in fourteen days nearly cured, though still some movement.

*Case* 9.—A. P., aged eleven, female. Third attack. The first occurred one year, the second five months, previously. Had been ill this time one month. The right side was principally affected. No dysphagia or difficulty of speech; tongue steady; general aspect healthy; bowels regular; appetite good; heart normal; never had rheumatism. She took zinci sulph. gr. ij. ex infus. calumbæ ℥j. t. d. for four days; then gr. iv. for twenty days, with daily shower-bath. Presented cured on the twenty-fourth day. When in the hospital five months previously she had been treated by syrup. ferri iod. ʒss. t. d. for thirty-one days, and went out cured on the thirty-second day.

*Case* 10.—R. H., aged eight, male. Received a "thrashing" from his father for letting fall some article, and immediately afterwards suffered from choreic spasm. Was unable to say whether a spasm caused him to drop the vessel. This occurred four months previously. The attack had not been very severe, most so on the right side. Speech, sleep, and swallowing were unaffected. The tongue was protruded fairly. General health good. Never had rheumatism or cardiac symptoms. Some flying pains, apparently of a neuralgic character. Took zinci sulph. gr. ij. ex infus. valerianæ ℥iss. t. d. for seven days, and gr. iv. for twenty-two days, with daily shower-bath. Improved steadily. Presented cured on the twenty-ninth day.

*Case* 11.—H. C., aged eleven, female, at school. Had been ill three weeks previously, and had partially recovered. Ten days back had again become worse. It was stated that the illness consisted of pains and swelling in the feet and arms, with tenderness, the child being at the same time feverish and out of sorts. It was not considered as rheumatism, and had no medical attention. Ten days before admission, the febrile symptoms recurred, with pain in all the limbs. Choreic movements were first noticed in the left side four days before, and had interrupted the sleep at night. They may have been caused by a fright which she seems to have had on that day. When seen, the child was sensible and intelligent. The movements were active and constant. Speech was slightly affected, but the tongue was fairly protruded. Swallowing was not interfered with. There was no evidence of other disease. She took zinci sulph. gr. ij. ter die, ex infus. valer. for five days. The dose was then increased to gr. iv., and this was taken for twenty-one days, with some amendment, At the end of this time she was given a daily shower-bath and liq. arsenicalis ♏iv. ter die. This she took for six days, at the end of which she was presented cured, having been thirty-three days in hospital.

*Case* 12.—D. R., aged fourteen, male. Had had chorea two months; no previous attack; never had rheumatism. No cause assigned for the attack. On admission, the movements were extensive, involving many muscles of the trunk; he could, however, walk. Speech was indistinct, the tongue protruded rapidly, and swallowing difficult. As this proved a very obstinate case, a variety of treatment was employed. The sulphate of zinc was given in doses of gr. j. ter die, with inf. valerianæ. After four days the dose was increased to gr. ij.; again, after three days, to gr. iij.; again, after four days, to gr. iv., which dose was taken for three days. As there seemed little or no improvement, the ferri carb. saccharatum gr. x. t. d. was ordered; after four days increased to gr. xv; again, after a week, to gr. xx.; and after a like period to gr. xxv. After three days this was raised to ʒss., which was taken for four days. There was then very little, if any amendment, though the general health was not materially impaired. The liq. potassæ arsenitis ♏iij. bis die was then ordered, with aperients on alternate nights Tepid shower-baths were also given twice a week. This medicine producing no effect after fourteen days, the ferri carb. saccharatum gr. xx. t. d. was again given for fourteen days, followed by a repetition of the arsenic in the same form for seventeen days. The effect of opiates was then tried. Morphiæ hydrochl. gr. 1-6th omni nocte, increased regularly to gr. 1-3d for eleven consecutive nights, but without much effect. The morphia was then administered in the form of gr. 1-6th omni manè, with only partial success. The patient had now been in hospital from December 31 to April 10 without very great improvement. At this date, however, the ferri carb. saccharatum was resumed in addition to the opiate in the last form, and increased regularly during the month from gr. x. ter die to ʒss. ter die. The choreic symptoms at length began to give way, and on May 1 he was presented cured, having been 123 days under treatment.

*Case* 13.—M. F., aged ten, female, at home. Had recently had measles, from which she was only partially recovered. Ten days previously began to suffer from chorea. Never had rheumatism. No previous attack. Not a very severe case; swallowing unaffected, speech very slightly so; tongue protruded rather rapidly. After free purgation took zinci sulph. gr. j. ter die ex inf. valerianæ for three days; gr. ij. for four days; gr. iij. for three days; gr. iv. for four days. She was then removed from the hospital while in a fair way of recovery, having been under treatment sixteen days.

*Case* 14.—J. B., aged ten, female. Not much history, except that she was a delicate and passionate child, and had had chorea about a fortnight. There was constant motion of both trunk and extremities, but the case was not very severe. Tongue hastily protruded. At first took zinci sulph. gr. j. ex inf. valerianæ ter die for three days; then gr. ij. for four days; then gr. iij. for four days with some advantage. But thirteen days after admission she was attacked with varicella, which run its usual course, and was followed by some impetiginous irritation of the skin. These did not prevent steady recovery from the choreic symptoms. She was presented cured on the forty-ninth day.

*Case* 15.—S. L., aged ten, female. Had been ill four months with chorea following a fright. Had never had rheumatism. Heart normal; the symptoms were not very severe; could walk perfectly; speech and swallowing unaffected; tongue fairly protruded; bowels rather costive. At first was freely purged. Then took zinci sulph. gr. j. t. d. for three days; gr. ij. for four days; gr. ij. for seven days; gr. iv. for three days. During this time there was no great improvement. She was then ordered the ferri carb. saccharatum gr. x. ter die. This she took for four days. It was then increased to gr. xv. with the shower-bath daily; continued for seven days. Then increased again to gr. xx. which was taken for fourteen days. With this there was some amendment. She was then given vin. ferri ʒj. and ol. morrhuæ ʒj. bis die. With this she made a recovery, and was presented on the sixty-fifth day cured.

*Case* 16.—J. E. P., aged eleven, mále. Chorea a fortnight; not very severe; no previous attack; had rheumatic fever some time before. No cardiac affection; speech and swallowing unaffected; tongue fairly protruded. At first had only cold shower-baths daily for eight days. Then took liq. potassæ arsenitis ♏v. ter die for eleven days, and had a blister on the nape of the neck. With this there was some improvement. The zinci sulph. gr. ij. ter die was then given for seven days with great improvement. It was increased to gr. iij. for three days, and to gr. iv. for ten days, by which time the cure was effected. Discharged after being in hospital thirty-nine days.

Of the sixteen cases described above, five were males, eleven females. The ages of all are intermediate between eight and sixteen years. Previous attacks had occurred in two cases only. Duration of previous illness had been one week in two cases; two weeks in three; three weeks in three; a month in two; two months in two; three months in one; four months in two, and a year in one case. The attacks were without known cause in four instances, from fright in four, from a fall in one, and followed previous illness in five cases. This illness was in one instance of febrile character, probably scarlatina; in another measles, and in three others acute rheumatism; of these latter cases one also suffered from cardiac disease, ultimately terminating in death. Two other cases, though not absolutely complicated with illness, showed evidence of being connected with commencing menstruation in a feeble habit. One case was attacked with varicella soon after admission.

As a general statement few of the above cases were characterized by great severity, and of these, one, Case 12, which warrants such a description, was more remarkable for its obstinacy than for the acuteness of the symptoms. Accordingly, the graver disturbances of function are comparatively infrequent. Interference with articulate speech was only observed in six of the cases; dysphagia, to an extent beyond what would naturally follow from spasmodic contortions of the face and hands, only in one case. The rapid and spasmodic protrusion of the tongue, to which much attention has been drawn as a symptom of chorea, was observed in five cases. They formed the bulk of those which have already been mentioned for imperfection of speech. The one remaining case had recovered considerably from this latter symptom before admission, and it is probable that, at an earlier period, spasm of the tongue might also have been substantiated. Distinct spinal tenderness was only found in four cases.

Sulphate of zinc was alone exhibited in eight cases. Of these, five were cured; three were improved, though not cured. It was in all cases administered in increasing doses, beginning with one or two grains. Six grains was the highest dose given in the cases before us, though in others four times this amount has been exhibited with advantage, and without vomiting. The shortest effective administration was of twenty-four days duration among the cured cases; the longest of fifty-six days. The average period of continuing the medicine was twenty-nine days for all the cases; two of which were removed before its full influence had been produced, and one was interrupted by an attack of varicella. In four cases the sulphate of zinc was followed by ferruginous preparations. One of these, Case 5, complicated by rheumatism and cardiac disease, must be regarded as an exception. In the other three, Cases 1, 12, and 15, the remedy must be considered to have failed, or only to have succeeded partially; they were all ultimately cured under the administration of iron.

In four cases, the zinc was followed by a course of liq. arsenicalis. In three of these, Cases 3, 7, and 11, this effected a cure; but in the other, Case 12, it was equally ineffectual with the zinc, and recovery took place under the iron treatment. In Case 16, on the other hand, the liq. arsenicalis was exhibited for eleven days in doses of ♏v. ter die, without much effect, and recovery took place after the administration of sulphate of zinc for nineteen days in doses of gr. iij. ter die. In two cases, 5 and 13, opiates formed part of the treatment. The former of these required their use principally from rheumatic and cardiac complication; in the latter they seem to have had a beneficial effect; inasmuch as recovery took place under their use combined with iron, though the disease had previously resisted both remedies when exhibited singly.

Besides the principal remedy, thirteen cases had the cold or tepid shower-bath during the treatment. Of the three not so treated, one was confined to bed with subacute rheumatism and cardiac symptoms; the second had only recently recovered from an attack of measles; the third had varicella soon after admission. It is not possible to give any separate estimate of the value of this application in bringing about the cure, though it cannot but be ranked very high. It may be omitted as an element of comparison, from its employment in all cases which did not exhibit a distinct counter-indication.

The general statistics are as follows: Of 16 cases treated with sulphate of zinc, 13 went out cured, 3 relieved; but 2 of the latter were in a fair way of recovery, and may probably be set to the credit of the medicament. On the other hand, three of those ultimately cured owed their improvement partly to ferruginous preparations; and in one case the zinc had no effect whatever. It may, then, be stated generally, that advantage was derived from the zinc in 14 out of 16 cases. The longest stay in the hospital among these cases was 123 days; the shortest 14; the average stay, 44·6 days.

Fourteen cases were treated during the same period with preparations of iron; all were cured. The longest stay in hospital was 161 days; the shortest 6 days; average stay, 44·2 days.

Twenty cases were treated with liq. potassæ arsenitis: 18 cured; 1 relieved; 1 died. The longest stay in hospital was 55 days; the shortest 6; average stay, 26·3 days. Average stay in hospital of the 50 cases submitted to three principal remedies, 37·2 days; average stay of all the 54 cases, 35·4 days.

The results of this analysis are somewhat remarkable, as failing to confirm the usual estimate of the value of sulphate of zinc in this disorder. The iron seems to act more certainly, and the arsenic both more certainly and more rapidly than the zinc. The average duration of treatment both with iron and zinc, 44·2 days and 44·6 days respectively, is very similar, and both are above the general average of the whole number of cases, namely 35·4 days; whereas the average stay of the arsenic cases falls as low as 26·3 days. This difference is the more remarkable, as the character of the cases submitted to the arsenical treatment rather exceeded in severity that of the others; and, indeed, the only death recorded belonged to this division.

It remains a question whether the discrepancy between these results and those of some previous well-conducted observations is due to mere accident, or to real difference in type between cases originating at different times and under dissimilar circumstances.—(*Med. Times and Gazette*, Sept. 17, 1859.)

2. On the Hypodermic Treatment of Diseases. By *Charles Hunter, Esq.*, late House Surgeon to St. George's Hospital.—Medicinal substances may be introduced into the cellular tissue beneath the skin with the greatest facility by means of a minute syringe, and so introduced certain medicines will act with extraordinary rapidity, and the most satisfactory result.

To distinguish this plan of injecting beneath the skin from the epidermic and endermic, it would be as well perhaps to use similar phraseology, and call this the hypodermic method.

Dr. Alexander Wood, of Edinburgh, has long employed the introduction of medicines into and under the skin, in the various forms of neuralgia. To him is due the discovery of injecting narcotics into the tender spots of the neuralgic part or tissue, whichever it happens to be, the belief of Dr. Wood being that the *localization* of the remedy in the neuralgic part is the cause of the success which attends his practice.

I have tried Dr. Wood's treatment and localization of the narcotic injection to the neuralgic part, and found it productive of considerable relief; as, however, I found frequent repetition of the injection in the same spot productive of abscess, I employed the hypodermic injection of other parts of the body, avoiding localization of the injection; and I have found that the hypodermic injection of the cellular tissue beneath the cutis of any part of the body is quite as striking and as curative in its effects as the injection localized to the neuralgic tissue; moreover, it has these advantages, viz., that inflammation is less likely to follow, and less pain must necessarily accompany the injection of a sound than an unsound or morbidly sensitive part.

In the present communication I wish to draw the attention of the profession to this mode of introducing medicine as applicable in many other affections besides neuralgia.

How often we find that medicine fails to afford sleep to the restless, the highly excitable, the delirious, and the maniacal. In all these cases I have tried the hypodermic injection of narcotics, (often when other measures had been tried and failed,) and in all the effect was immediate, or nearly so, quiet or sleep directly supervening.

My proposition is: That to produce an immediate or decided effect no method is more effectual than the hypodermic injection of the cellular tissue.

In favor of this being the case, (venous injection being set aside on account of its danger,) I will relate some experiments, but must first state the reason the word "narcotic" is inserted in the proposition, is because narcotics are the class of medicines I have always employed in the cases in which I have tried the hypodermic injection, and they are the class of remedies called for generally where an immediate effect is required, either to relieve pain or produce sleep.

The following experiments will show with what rapidity medicines injected into the cellular tissue of the body will act:—

*Experiment* 1. Injected a few drops of water containing one-twelfth of a grain of the acetate of strychnia into the cellular tissue of a cat; in one minute it was tetanic, and in two minutes it was dead.

*Experiment* 2. I injected half a grain of morphia in the subcutaneous cellular tissue of a rabbit; it was completely comatose in five minutes, and remained so for hours.

*Experiment* 3. By the kind request of Dr. Page, I injected half a grain of the acetate of morphia into the arm of a girl aged sixteen, suffering from extreme chorea of all the muscles of the body; in two minutes all the muscles had ceased their irregular movements, and in four minutes the girl was asleep.

*Experiment* 4. A man suffering from tic douloureux was for some time subjected to the hypodermic treatment; he almost constantly slept in from two to three minutes after the dose was injected at night.

In several cats which I injected with morphia, the first symptoms showed themselves in a very few minutes.

From the following experimental observations on man and animals it will be seen that:—

1. Hypodermic injections act quicker than stomachic doses.

2. That they produce a more powerful, a more effective result.

3. That frequently the hypodermic injection is productive of the greatest benefit, while an equivalent dose administered by the stomach is entirely useless, and even prejudicial.

*Experiments* 5 *and* 6. I injected three-quarters of a grain of the acetate of morphia into the cellular tissue of a man with *mania a potu.* He got to sleep almost directly, and slept for many hours. I gave the same man the same dose (three-quarters of a grain of morphia) in one ounce of water as a stomachic dose; it was one hour before sleep was produced, which, when obtained, lasted only about one hour and a half.

*Experiments* 7 *and* 8. I obtained a fine healthy rabbit, and, assisted by Mr. Venning, injected half a grain of morphia in about three drops of water, into the subcutaneous cellular tissue of the animal. It appeared slightly affected in two minutes, and fell comatose, without a stagger, in five. The pulse and respiration were very much lowered at the same time; narcotism lasted in this rabbit from six to seven hours; it appeared perfectly convalescent in nine hours.

Some days after, Mr. Venning present, I again attempted to narcotize the rabbit by the same dose given by the stomach: a gum catheter was used to make certain of the fluid enter the stomach; for a long while we awaited the result, but no narcotism followed; the rabbit never even went to sleep, nor did it bring up the morphia.

*Experiment* 9. I injected three-quarters of a grain of morphia into the cellular tissue of a cat; it became preternaturally quiet directly after; in seven minutes it gave a convulsive start, its position previously having been fixed and uncomfortable; in thirteen minutes it uttered short sharp cries; muscles of body rigid; eighteen minutes, muscles of extremities become rigid also; the rigidity of the muscles became more marked, until at forty minutes it was in a general tetanic condition, the body fixed, and the legs extended. The cat, contrary to expectation, recovered; but for many hours suffered from the morphia, which appeared to throw the animal into a state of drunkenness, after the muscular rigidity had passed off. Two or three other cats, with a similar dose, I have thrown into a state of strong convulsion, and even of tetanic spasm, by the injected dose of morphia.

*Experiment* 10. With the aid of Mr. Keal I administered the same dose of morphia to the same cat as in Experiment 9, by the stomach, the gum catheter being passed down the œsophagus. The cat was quite natural for five minutes, then became quiet, and was sick in eight minutes, with five or six spasmodic retchings; it began the purring which morphia causes in all cats in fifteen minutes; at twenty minutes and thirty-five minutes it had one leg convulsed, and in one and a half hours became very excitable and constantly on the move, as if tipsy; this lasted several hours; but it never had the general spasms, or rigidity which the injected dose produced.

*Experiment* 11. With Mr. Ash, I gave the same cat the same dose by the stomach in the same quantity of water, but this time warm, (instead of cold, as in Experiment 10.) Sickness was again the first well-marked symptom of the action of the morphia; it took place in ten minutes; the succeeding symptoms were as in last experiment, but milder.

As the question might arise whether the sickness in these last two experiments might not have been due to the mechanical irritation of the stomach by the catheter, we made some experiments to set it at rest. We went through the process of injecting, at different times, cold and warm water into the stomach of the same cat, with the cathether; no sickness or irritation of any kind ensued.

*Experiment* 12. In a patient with hemicrania no narcotic except morphia, injected, will cause sleep. Opium and morphia have repeatedly been tried by the stomach; they never produce sleep, they always cause sickness.

From experiment we may gain much; but far more satisfactory is the proof if it be derived from cases in which the remedy has been called for, tried, and found successful, than from its trial on animals.

Besides such cerebral affections as delirium tremens and mania, in which I mentioned this treatment as producing instant effect and speedy relief, even where large quantities of narcotic medicines had failed by the stomach, there are cases of functional cerebral derangement of the milder form, manifested by wakefulness, continued perhaps for days together, accompanied, it may be or it may not be, by excitability.

I may refer to two cases of this kind in which I have employed this treatment of narcotic injections with marked and immediate effect; and it may be observed that, in both, the administration of narcotics by the stomach *had failed.*

CASE VII. *Continued Wakefulness, with Excitement.*—James H., aged about 40, a man of temperate habits, of a highly nervous temperament, was admitted into St. George's Hospital, in December last, under the care of Mr. Tatum. He was suffering, on admission, from a gunshot wound of the foot, from which part the bullet was extracted the day he came in, while he was under the influence of chloroform. The wound caused the patient to be in a perpetual worry and anxiety about himself, which at last got to such a pitch that he could get no rest. After two days of wakefulness, during which time he was perpetually talking and getting more excited, and could neither be quieted by morphia, opium, nor hyoscyamus—the opium, on the contrary, causing a little delirum—I injected,*

* The instrument I employ is the same in construction as the syringe for injecting aneurisms, with a gold nozzle, sharp-pointed and perforated. The makers of this syringe, (which works by a screw, each whole turn of which is equivalent to one minim.) and of a cheaper one, without the screw, but graduated, are Wicker and Blaize, St. James Street.

at the request of Mr. Tatum, half a grain of the acetate of morphia into the cellular tissue of the right arm. At the time I injected the morphia, the man was talking in an excited manner, and did not feel the point of the syringe which I quickly thrust beneath his skin; this was at 9.30 A.M.

In *five* minutes he was asleep, and with the exception of a few minutes, about 11.30 P.M., he slept the whole of the night. The benefit this patient received from the injected dose of morphia did not end with the sleep; he was much quieter for the next few days, his appetite returned, and his face lost the flushed and anxious look it before had. The injection was not again required.

Case VIII. *Wakefulness without Excitement.*—This case was also under Mr. Tatum, in St. George's Hospital. Mary J., aged 35, of a hysterical aspect, was admitted for some subacute affection of the hip. For nights together this woman never slept, (carefully watched by the night nurse,) and morphia, in doses up to three-quarters of a grain by the stomach, produced no effect at all, whereas the same doses injected beneath the skin would cause her to sleep soundly.

From purely cerebral cases I now pass to one in which it seemed the spinal cord was also affected, judging from the constant and violent spasms of the muscles.

Case IX. This patient was a girl, Mary D., aged 16, admitted November 26, under the care of Dr. Page. On admission she was in a frightful state of chorea, all the muscles of the body being perpetually in action.

December 7 she was still in the same state, no treatment apparently doing any good. The chorea was constant, day and night, and the movements were so violent that she could hardly at times be kept in bed. At this time she was much more emaciated than when she came in, and sordes were beginning to collect about the lips; she had not slept for three days or nights; and it seemed now that if sleep was not procured she must die of exhaustion.

At 11 P.M., the movements of the arms being for a moment restrained, I injected one-third of a grain of morphia into the cellular tissue of the neck near the clavicle.

From that moment the violence of the spasms gradually abated, and in fifteen minutes she was in a sound sleep, and snoring. The chorea completely stopped the moment she slept. In three-quarters of an hour she woke up, and was then as bad as before.

At half-past 2 A.M. I injected half a grain of morphia, as the effects of the preceding dose had completely gone off some time; muscular action quieted instantly; she was dozing in two, and slept in four minutes; she continued to sleep three hours.

On December 8 the chorea was as bad as ever. Sordes about the lips increased; pulse very weak. In the evening I injected one-third of a grain of morphia; she dozed in seven minutes and had a little sleep, and the spasms were quiet for half an hour after it. At 1 A.M. I injected half a grain, and, as in the preceding night, she was asleep in four minutes, and did not wake for six hours and a half.

After the 8th I found that the introduction of half a grain of morphia into the arm would always and instantly quiet the spasms for several hours; and three-quarter grain doses would give her sleep. For a few days she considerably improved, evidently owing to the sleep she obtained, and the cessation and quieting of the spasms; she took food better, and the sordes on the lips disappeared; but she ultimately died from exhaustion, and rapid wasting accompanying the formation of foul abscesses in the cheek and throat.

CASE X. *Tetanus.*—William D., aged 60, was admitted in St. George's Hospital, November 5, 1858, with a gunshot wound of the thigh. On the 13th, he had a little wandering delirium; on the 14th, trismus and other symptoms of tetanus supervened, and gradually increased during the two following days.

16th. Has had no good sleep since the spasms came on. At half-past twelve three-quarters of a grain were injected in five drops of liquid; he was asleep in twenty-five minutes, and remained asleep for the greater part of the night, totally undisturbed by the tetanic spasms, which recurred nearly every minute the whole time he was asleep.

The progress of the case did not appear to be affected by this treatment, which certainly procured sleep, but had no effect upon the spasms, from which death finally resulted.

*Remarks.*—In Cases IX. and X. it is interesting to observe, that sleep followed the injection of the narcotic, but that the effect on the spasms was not the same in both; for instance, in the girl, the dose being injected, muscular action instantly became more quiet, and sleep generally resulted in about four minutes, no spasms taking place while the sleep lasted; and, in all probability, life was in her prolonged by the sleep obtained, and the quieting of the spasms. On the contrary, in the man with tetanus, although the brain was speedily quieted, and sound sleep of some hours procured, no effect at all was produced on the spasms, which recurred every minute during the night. May not the persistence of muscular action during sleep be a guide as to what spasms are cerebral and what spinal—especially with regard to chorea, looked on by some as an affection purely of the spinal marrow?

In such cases as tic douloureux, sciatica, and constant or occasional pains of the nerves, a cure may follow a single injection, or if not, more or less benefit will in all probability be effected. I have had no case in which some benefit was not produced. But from the cases of this description that I have had under my care, I cannot find that localization of the injection adds at all to the cure, except in those cases in which the mind of the patient has something to do with it, and there is a confidence alone in the local treatment. From the following case the non-necessity of localization will be made apparent.

CASE XI. *Sciatica.*—In this patient the sciatica was in the left leg, but all the injections were made in the cellular tissue of the right arm. J. D., aged 70, was admitted November 24, 1858, in St. George's Hospital, under the care of Dr. Page. He had been subject to gout, and for a fortnight, before and after admission, suffered day and night from pain in the region of the left sciatic nerve. He was treated with morphia and colchicum, and then with salines and hyoscyamus, without the least good effect.

December 6. I injected half a grain of morphia into the right arm at 9.30 P.M. At 9 45 I returned to the ward he was in, and found him sound asleep; and he slept all night.

7. He has no pain at all in the leg.

11. Still has no pain in the leg; he is not now restless at night but sleeps well.

12. The pain has come back again, and owing to it he had no sleep last night. I injected half a grain of morphia again in the arm; he lost the pain immediately, and was asleep in a few minutes, and had a "beautiful night."

18. Has again had no pain for six days, till to-day; injection repeated in the arm.

This patient, from having the pains day and night, was made "well," to use his own expression, for seven or eight days together, by a single injected dose of morphia, whereas narcotics by the stomach had done no good.

Case XII. *Sciatica.*—Another patient with this affection, on whom, by Dr. Page's kindness, I tried this treatment, went out cured after five injections. This patient had also taken colchicum, morphia and other medicines, by the stomach, without effect. With regard to the part injected, I twice localized the treatment in the sciatic region, and three times injected the arm, the patient each day becoming freer from pain, and stronger in his walk.

I understand that the sciatica in this patient has since returned, but not so badly as to occasion him to become an in-patient of the hospital.

*Tic Douloureux.*—In this affection I believe that great benefit, if not a cure, generally attends the hypodermic introduction of narcotics; but that the localization of the treatment to the neuralgic part is necessary, I do not believe, as I stated in this journal six months ago, nor have I since then found reason to change my opinion. The following case is to the point:—

Case XIII. *Tic Douloureux.*—A lady, suffering acutely from neuralgia of many months' standing, sent for me to employ this treatment. She had taken hyoscyamus, opium, and morphia, internally, with no effect on the neuralgia; but they all, more or less, affected her head. She had also tried the endermic application of morphia. She found the endermic method, which was employed over the part affected, give her more relief than the administration of the narcotic by the stomach; but the effect lasted only a few hours, and she was as bad as ever the next day. The situation of the tic was the side of the face and head.

I injected a little more than half a grain of morphia into the arm; giddiness came on in three minutes, followed by considerable sickness, and subsequently by sleep. The pain ceased at the time, and has never since returned;* it is now between three and four months since the dose was injected.

---

* In Edinburgh chiefly, and also in London, I am told that the sickness following the injection of narcotics is often excessive, and has, consequently, caused the treatment to be less adopted than it otherwise would. In all the cases in which I have employed the narcotic injection I have only once found urgent sickness produced, viz., in the case given above; and here the cause of sickness may partly be explained thus: that the patient was a woman of highly nervous temperament, and badly affected by narcotics, however administered. The amount injected has also something to do with it. In the usual way I think the stomach is saved disturbance and irritation by the injection of the cellular tissue, (which is the part I always inject;) and for these reasons: 1. That I have so seldom found sickness at all as a consequence. 2. Because in one patient accustomed to 30 or 40 grains of morphia daily for years, the tympanitic and disordered state of the stomach recovered itself while the hypodermic injection was gone on with, and which never once caused sickness. 3. Because, in one well-marked case. all narcotics administered by the stomach caused sickness, which was never the consequence of an injected dose; which latter, if small, quieted, and if large, caused good sleep.

The non-localizing plan has this advantage, that no abscess results in cases where several injections are required; for example, in a patient with severe neuralgia of many years' standing, I at one time employed the injection three times a day, varying the site each time. The patient was cured, and had no abscess whatsoever in the parts injected. Another patient, with tic douloureux, who *would* have the injection employed locally, had considerable tenderness, and some inflammation, as the consequence. The non-localizing plan, employed subsequently, caused no irritation.

It may be observed, that in the cases recorded I have always employed morphia as the narcotic to inject; I have employed it far more frequently than any other; but have also used the following, and chiefly in neuralgia:—

Evaporated tinct. of opium; tinct. aconite; tinct. cannabis indica; concentrated liquor opii sedativus, (prepared by Bullock;) sulphate of atropine, and chloroform.

The solutions I prefer are those of morphia and atropine, and the evaporated tincture of opium, (from one part to a third or fourth,) by which means the spirit is got rid of.

How it is that these narcotics are so rapidly and effectually injected into the cellular tissue, I intend considering at some future time, and also to bring before the profession the result of further experiments on animals with other anodyne and anæsthetic agents.—(*Med. Times and Gazette*, March 5, and April 16, 1859, pp. 234, 387; *Braithwaite's Retrospect*, part 39, p. 56.)

3. ON SOME POINTS IN THE TREATMENT AND CLINICAL HISTORY OF ASTHMA. By *Hyde Salter, M.D., F.R.S.*, Assistant Physician to Charing Cross Hospital.—One of the commonest and best-reputed remedies of asthma, one that is almost sure to have been tried in any case that may come under our observation, and one that in many cases is more efficacious than any other, is strong coffee. To the question, "Have you tried strong coffee?" the asthmatic is pretty sure to answer "Yes:" and he is also pretty sure to add that it gives him relief.

About the *modus operandi* of this remedy I was long puzzled; I could not make it out; and it is only lately that I think I have stumbled upon it. The *rationale* of its efficacy is, I think, to be found, on the one hand, in the physiological effects of coffee,—the particular nervous condition that it produces,—and, on the other, in a feature in the clinical history of asthma which I have long observed, and of which I think the efficacy of coffee is highly corroborative.

This fact is, that sleep favors asthma—that spasm of the bronchial tubes is more prone to occur during the insensibility and lethargy of sleep than during the waking hours, when the senses and the will are active. I have already referred to this in my observations on the "Clinical History of Asthma," in explaining why the paroxysm invariably (or almost invariably) chooses the hours of mid-sleep for its onset. Let me just refer to this subject again; for it is both interesting and important, as it explains a curious and very constant phenomenon in asthma—the hour, namely, of the attack—is highly illustrative of its pathology, and furnishes the key to some of its treatment.

I think, then, that sleep favors the development of asthma in two ways—

1. By producing insensibility to respiratory arrears.

2. By exalting reflex action.

The way in which sleep favors the development of asthma, by producing insensibility to respiratory arrears, and exalting reflex nervous action, I have already sufficiently explained in the papers on the Clinical History of Asthma to which I have referred.

There can be no doubt that sleep does exalt reflex nervous action. It is a fact so abundantly inculcated by the history of disease as hardly to require illustration or proof. The phenomena of epilepsy, cramp, lead tremors, and other examples of deranged muscular action, all teach it. It is just as sleep comes on, just as the will is laid to rest, or during sleep, that these different forms of involuntary muscular contraction most commonly occur. Any one, to convince himself of it, has only to fall asleep sitting on the edge of his chair, in such a position that it shall press on his sciatic nerves. As long as he is awake his legs will be motionless; but the moment he falls asleep they will start up with a plunge and suddenly wake him. As soon as he is awake they are quiet and still again, with no disposition to start, till he again falls asleep, and that moment they start again and wake him; and so he may go on as long as he likes. He changes his position, sits back in his chair, and they start no more. I need not explain what so clearly explains itself. I heard, some years ago, of a case of what might be called chronic traumatic tetanus, in which the source of irritation —the excito-motory stimulant—was extensive disease of the hip-joint. The moment the patient fell asleep he was seized with opisthotonos, which, of course, immediately woke him. On awakening, the tetanus vanished; on again falling asleep, it reappeared; and this alternation of falling asleep and waking continued for weeks, if not for months, the patient getting no continuous rest, till he was quite worn out. As long as he was broad awake the tetanus never appeared.* Hosts of similar facts, illustrative of the same truth, might be cited.

Anything that exalts reflex nervous action increases, of course, the potency of reflex stimuli. Now, I have elsewhere endeavored to show that the phenomena of asthma are, in almost every case, those of excito-motory action, and that the exciting causes of asthma are, in the great majority of instances, such as act by a reflex circuit. They would, therefore, on the asthmatic's falling asleep, immediately acquire a potency they did not before possess, just as the pressure on the sciatic nerve did, in the illustration I have given. Thus it is we see that the asthmatic may gorge himself with unwholesomes, and yet, as long as he keeps himself awake, suffer no consequential asthma;—the irritant is there, the undigested food is in the stomach, but as long as he is awake, as long as the will is dominant, it is inadequate to the production of reflex phenomena. But let him fall asleep, and in an hour or two the paroxysm will be established.

And not only will sound sleep determine, by this exaltation of reflex susceptibility, the production of asthma by its exciting causes, but a small dose of the same condition—sleepiness, drowsiness—will favor the supervention of asthma in a proportionate degree. Not only is drowsiness a premonitory sign of an

* I was further informed, respecting this case, that, after everything else had failed, sleep was procured, with an immunity from the tetanic spasms, by putting the patient into the mesmeric state. In this way he got rest, and greatly improved; but what was the ultimate issue of the case I do not know.

attack, but a powerful predisposer to it; and the asthmatic knows that he yields to it at his peril. I have often noticed in asthmatics that the sleepiness that is so apt to come on after dinner will be accompanied by a slight asthmatic oppression and wheezing: as the drowsiness deepens, so does the asthma, and in this way it may settle down into an attack; but if the patient rouses himself, or if anything occurs to engross his attention so as to wake him up, broad awake, the asthma quickly vanishes. It is in this way, I think, that is to be explained the fact that asthmatics can dine out late and unwholesomely with impunity; while, if they dine at the same time and the same way at home, asthma is sure to come on. At home they want that excitement which at a dinner-party keeps the animal functions in a state of exaltation and the mind vividly awake, and effectually banishes the least approach to drowsiness. Of the fact there is not the slightest doubt. I know an asthmatic who can with impunity dine out at seven o'clock, as dinner-eaters of the nineteenth century are apt to dine—shirk nothing from soup to coffee—walk home at ten o'clock, a distance perhaps of four miles, with the wind of a deer-stalker—go straight to bed, and get up the next morning scathless; but if he were to dine at home at six, or even at five o'clock, he would be wheezing at nine, and by four the next morning downright asthmatic.

I believe a certain amount of the curative influence of fright, or other strong mental emotion, is to be explained in the same way.

"But why," it may be asked, "all this roundabout digression? What has all this to do with the curative influence of coffee?" I believe it is simply its explanation. For, what are the physiological effects of coffee? They consist in the production of a state of mental activity and vivacity, of acuteness of perception, and energy of volition, well known to those who have experienced it, and to a certain extent very pleasurable, and which is the very reverse of that abeyance of will and perception which, in drowsiness or sleep, so favors the development of asthma. In sleep, will and sense are suspended; after taking strong coffee, they are not only active, but exalted. It produces rapidity of thought, vivacity of spirits, clearness of apprehension, increases tenfold the working powers, and altogether intensifies mental processes. Not only is there no disposition to sleep, but sleep is impossible: the thoughts hurry one another through the mind; the bodily movements are energetic and rapid; and if the effects of the drug are pushed far, a very unpleasant condition is produced, something like that of delirium tremens, *minus* its hallucinations. Now, if the suspension of the will, or its depression, favors the production of excito-motory phenomena, and thus favors the development of asthma, is it unreasonable to suppose that its exaltation should prevent or cure it? It must do so—if not positively, at least negatively, by removing the predisposing condition. And bearing in mind this marked physiological effect of coffee—that this exaltation of the animal nervous function is exactly what it produces—it certainly does seem to me reasonable to suppose that this is its *modus operandi*. And if of coffee, then of strong tea, and alcohol, and ammonia, and ether, and other stimulants of undoubted value in asthma.

To show that this is the *rationale* of the cure of asthma by stimulants, I do not think it is necessary to show that it is only when the asthmatic is drowsy, or has been sleeping, that they do good. If anything that rouses the asthmatic to a state of wakefulness will put a stop to asthma that was creeping on him while he was sleeping or sleepy, *a fortiori* anything that carries him beyond a state of mere wakefulness—that gives him an active, not a mere passive wakefulness, will be still more efficacious, and will be adequate to the checking of an attack that, in spite of his being broad awake, was gaining on him.

The very frequency with which coffee gives relief, makes it hardly worth while for me to narrate the history of any cases. I should think, from my own experience, that coffee relieves asthma in two-thirds of the cases in which it is tried. The relief is very unequal, often merely temporary, and sometimes very slight: sometimes it is complete and permanent. It is often taken in the morning; and patients will tell you, that previous to taking their coffee they are not fit for anything—can hardly move about; but that taking it is immediately followed by freedom of breathing, and an ability to enter at once on their daily occupations.

There are two or three practical hints with regard to the administration of coffee that are worth bearing in mind.

1. It cannot be given too strong. Unless sufficiently strong to produce its characteristic physiological effects it does no good, but rather harm; moreover, if given very strong it need not be given in much bulk, and quantity is a disadvantage—its effect is less rapid and it oppressively distends the stomach.

2. I think it is best given without sugar and milk—pure *café noir.*

3. It should be given on an empty stomach; if given on a full stomach it often does great harm, by putting a stop to the process of digestion: indeed, so much is this the case, that I consider coffee accompanying a meal, especially late in the day, so peculiarly apt to induce asthma that it deserves to be classed among its special provocatives. I have mentioned elsewhere the case of an individual who never dared to take the usual after-dinner cup of coffee—it would make the simplest dinner disagree with him. But the same asthmatic found in strong coffee, on an empty stomach, one of his most valuable remedies.*

4. For some reason or other, I don't know why, it seems to act better if given hot—very hot.

I adverted just now to the influence of mental emotion on asthma, and stated my belief that its *modus operandi* was, like that of coffee and other stimulants,

---

* Since writing the above I have received the following account, from an asthmatic gentleman, singularly confirmatory of my own observations:—

"I used to think," writes my informant, "strong coffee the best of all remedies. I remember one instance especially, only a pattern of many others, but more striking when told. With bent back, high shoulders, and elbows fixed on the chair-arms, I had been laboring for breath all the afternoon. About five o'clock I had two breakfast-cups of strong coffee. The hard breathing disappeared rapidly and completely. My sisters were dancing in the next room, and in less than an hour I was dancing with them, quite free from asthma.

"Of late, coffee has often had an opposite effect upon me. The after-dinner cup of coffee, to which I have been for several years habituated, now produces a sensation of stuffing of the chest, and incapacity of moving about. I believe this is because it stops digestion; and the reason I did not suffer for some years I take to be, that my originally most excellent and enduring stomach could stand it so long, and no longer. Coffee, on an empty stomach, I still deem a most valuable remedy. I do not share the prejudice against putting milk and sugar into coffee that is used as a medicine, provided that it remain *café noir*, and be not made *café au lait.*"

by producing an exaltation of sense and will—an intense activity of the intellectual part of nervous action—and proportionately lessening the tendency to excito-motion; and this it does to a much greater degree than stimulant remedies, and its effects are, therefore, proportionately more sudden and complete. It was, indeed, the curative influence of violent emotion, and the observation that it and coffee taking alike banish that condition in which asthma is most prone to come on, that first suggested to my mind the theory of the action of stimulants on asthma that I have just endeavored to propound. I think, too, that mental emotion acts, if I may so express it, as a nervous derivative. There are many phenomena, both in health and disease, that seem to show that only a certain amount of nervous activity can be in operation at a certain time, and that if a nervous action of one kind comes into operation, another that had been previously going on is immediately depressed or arrested. Such is the explanation of the well-known experiment of the two dogs, one of which was taken hunting immediately after a meal, while the other was allowed to sleep. In the one that was taken hunting, digestion, on its return, was found hardly commenced; in the other, it was completely over, and the stomach empty. In the sleeping dog the whole vital dynamics, not being otherwise employed, were appropriated by the function of digestion; while in the hunted dog they were entirely taken up by its energetic locomotion, and drafted away, as it were, from that nervous superintendence of digestion without which the function cannot be carried on.* The power of strong emotion, or hard study, in retarding digestion, is an analogous fact. Just in the same way, I think, the extraordinary activity and exaltation of thought and perception that characterize the state of mind that the taking of coffee, ether, and other stimulants produces, acts as a nervous derivative in asthma, and diverts from the nervous system of the lungs that morbid activity which engenders the spasm of the bronchial tubes.

The cure of asthma by violent emotion is more sudden and complete than by any other remedy whatever; indeed, I know few things more striking and curious in the whole history of therapeutics. The remedy that stands next in speed and efficacy—tobacco pushed to collapse—takes time, a few minutes at least: but the cure of asthma by sudden alarm takes no time; it is instantaneous—the intensest paroxysm ceases on the instant. This is a fact so little known, as far as I can see, and yet so practically important and theoretically interesting, that I think it will not be unprofitable if I endeavor to impress it more deeply by the narration of some cases of its occurrence.

CASE I.—A gentleman suffering an unusually severe attack, so bad that he had been unable to speak or move all day, was suddenly alarmed by the illness of a relative: he ran down two flights of stairs and up again, and administered the restoratives he had procured, and then observed, to his astonishment, that his asthma was gone. This gentleman tells me that on many other occasions different forms of mental emotion have cured his asthma.

CASE II.—C. R., a confirmed asthmatic, states that when he was suffering from an unusually severe attack, a fire occurred just opposite his house. Pre-

* See Dr. John Reid's experiments, in Todd's Cyclopedia of Anatomy, vol. iii. p. 899; also those of Bernard and of Bischoff, in Müller's Archiv., 1843.

vious to the occurrence of the fire he was in bed, breathing with the greatest difficulty, and unable to move. When the excitement of the fire was over, he found that he had been standing in his night-shirt, looking, with the rest, out of the window, and that he had quite forgotten all about his asthma. His breath was not quite well the rest of the day, but nearly. On another occasion, when he was suffering from an attack, some sudden anxiety arose about two of the members of his family being out late: the alarm from which he suffered relieved his asthma, but not so suddenly as in the case of the fire. On another occasion, a sister of his was seized with sudden illness that seemed to threaten suffocation: he was suffering severely from asthma at the time, and was in bed; he jumped out of bed in great alarm, and found then that his asthma was perfectly cured. He was sufficiently well to run for a doctor, and continued well throughout the day.

CASE III.—Not long ago I was informed by a patient at the hospital, who had suffered greatly for many years, that however severe an attack might be, venereal excitement would almost invariably cure it. He told me also, that, when a youth, he had been guilty of the practice of onanism, and that the unnatural excitement thereby produced had just the same curative effect on his asthma, Indeed, he pleaded this effect of it as a sort of excuse for the practice; and assured me that when his breath was very bad at night he used to resort to it for the purpose of curing it.

I have known two or three cases in which sexual excitement has had just the same effect.

CASE IV.—The following account of the curative influence of mental excitement I have received from a medical friend, who has suffered from asthma all his life:—"On one occasion I was sitting with fixed elbows on a sofa, breathing hard: a lady came into the room whom I had known very well, and whom I had not seen for several years. I got up to receive her, and sat down again on a music-stool; with no special purchase, therefore, for the respiratory muscles, and yet with comparative ease of breathing. This ease lasted for about an hour, and then the difficulty of breathing come on again. I attribute the temporary amendment to the diversion of nervous energy. Just the same thing has happened to me more than once. On another occasion I was suffering a good deal at a farm-house. I got on horseback with some difficulty, and an anxious hope that the horse would go quietly, to fetch myself an emetic from a town three miles off. The horse ran away with me. I pulled in, at first weakly and almost despairingly, but the need of exertion brought the power: after a run of about a mile I succeeded in pulling up, and was delighted to find my asthma gone.—Another time I was breathing very hard, and a friend engaged me in an argument. At first I could only get out a sentence in successive gasps; but gradually, as I got excited, the hard breathing went off, and I could talk fluently."*

From the foregoing observations, then, I think we may conclude—

That, since the abeyance of the will favors, in proportion to the degree of that

---

* For additional cases of the cure of asthma by mental emotion, I must refer the reader to a paper on the Pathology of Asthma, in the British and Foreign Medico-Chirurgical Review for July, 1858.

abeyance, the development of asthma, and since the effect of strong coffee is to dispel such suspension or depression of volition, and restore the will to its wonted (or even an unwonted) activity, it is by thus exalting the will, and so disfavoring the development of excito-motory action, that this remedy relieves asthma.

That the same interpretation applies to the relief of asthma by all other stimulants whatever.

That thus strong coffee and mental excitement, although apparently so different, belong to the same category of remedies for asthma.—(*Edinburgh Medical Journal*, June, 1859.)

4. A CASE OF HÆMOPTYSIS, ENTRANCE OF AIR INTO THE VEINS AND DISCHARGE OF AIR BY A VENESECTION. By *M. Piédagnel*, Physician to the Hôtel-Dieu. (*L'Union Médicale*, 1859, No. 45.)—A gentleman, aged forty-two, of vigorous constitution and strong muscular development, had been affected for four years with a spinal disease, which, however, disappeared under treatment. For two months before coming under treatment he had influenza, with much cough, and occasional violent efforts at expectoration. On the 18th of February, 1858, while coughing, he suddenly fell down insensible, and discharged a considerable quantity of blood. The hemorrhage ceased, but consciousness did not return. When seen by M. Piédagnel he was lying on his back, perfectly insensible, face pale, eyes immovable, pupils the same, but distended; hearing gone; no movement or sensibility. The whole skin pale, and insensible to stimulants. Respiration noisy, but does not resemble that of cerebral congestion, being active in inspiration, and at the end of expiration as in very feeble children. There was a slight râle on the right, but a strong and very moist one to the left; the percussion was less clear on the left, but no dullness either before or behind. Percussion of the cardiac region only causes a doubtful dullness; on auscultation a dull, but tumultuous sound of the heart-beats was heard. The radial arteries were imperceptible; all the subcutaneous veins were empty. The diagnosis was doubtful; it could not be apoplexy; it might be laceration of the veins or rupture of the heart.

A variety of stimulants were applied; after about half an hour there were symptoms of returning animation; the cutaneous circulation reappeared. A venesection being proposed by M. Vivier, was performed on the median-basilic vein. A little blood dribbled out; to the great surprise of the by-standers bubbles of air were then seen to issue from the opening in the vein, at first one, then several, passing put so as to form a sort of wreath on the skin, between the opening in the vein and the lower part of the forearm. On the blood and the air ceasing to flow, some light frictions along the course of the vein caused a new issue of air-bubbles; two, four, eight, issued successively, then the flow stopped; the frictions were repeated several times; all precautions were taken to avoid error, and each time the same result ensued. At last blood and air ceased to appear; the patient did not improve, and death took place soon after. No autopsy was allowed. But the physicians were of opinion that a rupture of the lung had taken place, causing an entrance of air into the blood-vessels.—(*British and Foreign Medico-Chirurgical Review*, July, 1859.)

5. ON PUNCTURE OF HYDATID CYSTS OF THE LIVER WITH THE CAPILLARY TROCAR. By *Dr. J. Moissenet*, Physician to the Lariboisière Hospital. (*Archives Générales*, Fevrier, Mars, Avril, 1859.)—Having had the misfortune to lose a patient affected with a considerable hydatid cyst of the liver by peritonitis, resulting from a palliative puncture with the capillary trocar, the author enters upon a minute inquiry relative to the different methods which have been employed for the purpose of evacuating the liquid contents of the tumor and the subsequent destruction of the hydatids. He finds that experience justifies the simple puncture, provided there is no escape of the fluid contents into the peritoneal cavity. Récamier, Legroux, and Laugier, Owen Rees, Aran, Boinet, Robert, Cloquet, and others, have obtained successful results by puncture with a fine trocar. Cruveilhier, in speaking of Récamier's practice, warns against its general employment, unless adhesions can be proved to exist and the tumor presents a decided tendency to push outward. Dr. Moissenet brings forward several other cases besides his own which proved fatal. The first series of general conclusions that his analysis brings him to are:—

1. That the hydatid liquid, whether limpid or puriform, when poured into the peritoneum, whether as the result of accident or of an operation, induces acute or chronic inflammation, which is almost always, if not invariably, fatal.

2. That capillary puncture, though commonly not injurious, may induce effusion into the peritoneum of hydatid fluid, when there are no adhesions between the cystic and abdominal parietes; and that this effusion has taken place when the puncture has been made for exploration or palliation only; that is, when the cyst has been imperfectly emptied.

3. That the puncture of hydatid cysts, whether made with a capillary or an ordinary-sized trocar, may prove fatal by inducing inflammation of the cyst itself.

The second series of conclusions drawn by Dr. Moissenet are:—

1. That capillary puncture of a hydatid tumor, made even without the existence of adhesions, may be curative, when followed by as complete an evacuation of the liquid as possible.

2. That this result may be obtained by a single puncture, or by two or three successive punctures.

3. That the treatment commenced by capillary puncture must sometimes be completed by another method, as in the case of Dr. Owen Rees,* in which a larger trocar was used at the third puncture, and a gum-elastic sound left in the orifice.—(*British and Foreign Medico-Chirurgical Review*, July, 1859.)

---

* Guy's Hospital Reports, vol. vi., October, 1848.

